# Characterization of Inclusion Size Distributions in Steel Wire Rods

**DOI:** 10.3390/ma15217681

**Published:** 2022-11-01

**Authors:** Pablo Huazano-Estrada, Martín Herrera-Trejo, Manuel de J. Castro-Román, Jorge Ruiz-Mondragón

**Affiliations:** 1 Centro de Investigación y de Estudios Avanzados, CINVESTAV Saltillo, Av. Industria Metalúrgica No. 1062, Parque Industrial Saltillo-Ramos Arizpe, Ramos Arizpe 25900, Mexico; 2 COMIMSA, Saltillo 25290, Mexico

**Keywords:** inclusion, size distribution, population density function, extreme value theory

## Abstract

The control of inclusions in steel components is essential to guarantee strong performance. The reliable characterization of inclusion populations is essential not only to evaluate the quality of the components but also to allow the use of analytical procedures for the comparison and discrimination of inclusion populations. In this work, inclusion size distributions in wire rod specimens from six plant-scale heats were measured and analyzed. For the measurements, the metallographic procedure specified in the ASTM E2283 standard was used. The population density function (PDF) approach and the extreme value statistical procedure specified in the ASTM E2283 standard were used to analyze the whole size distribution and the upper tail of the size distribution, respectively. The PDF approach allowed us to identify differences among inclusion size distributions and showed that new inclusions were not formed after the liquid steel treatment process. The extreme value statistical procedure led to the prediction of the maximum inclusion length for each heat, which was used for the statistical discrimination of heats. Furthermore, the estimation of the probability of finding an inclusion larger than a given inclusion size using the extreme value theory allowed us to order the heats for different critical inclusion sizes.

## 1. Introduction

Control over inclusion cleanliness in steel products is necessary to ensure their performance under specific conditions. Inclusion size distribution is one of the parameters used to evaluate steel quality, and therefore its control during the steelmaking process is required [1,2]. Hence, the successful control of the inclusion size distribution must be based on analysis procedures that provide information on the formation and evolution of inclusions. Furthermore, predictive and discriminative analysis procedures are desirable.

The size distribution of an inclusion population is frequently estimated from metallography measurements and image analysis and represents an inclusion cleanliness parameter. Higgings [3] represented the particle size distribution by the population density function (PDF) as an alternative to the classic histogram format. The PDF approach presents the advantage of being user-independent compared to the histogram and the corresponding density function, which assumes a normal distribution [4]. Furthermore, the PDF is unique for a given inclusion population, provides information on the formation and evolution of inclusions [5] and enables the comparison of size distributions from different specimens. The logarithmic representation of PDFs is linear or quadratic and can be described by fractal (power law) and lognormal distributions, respectively [6]. Zinngrebe et al. [7] introduced the PDF approach in the analysis of inclusion size distribution and used it to study the formation of inclusions during the secondary steelmaking and casting process of Ti-alloyed Al-killed steel. The logarithmic representation of PDF showed a quadratic shape just after Al addition and became linear with time. A quadratic shape characterizes the transient deoxidation process where the transfer of the matter occurs between steel and inclusions, i.e., the formation and growth of inclusion process, whereas a linear behavior indicates that the equilibrium inclusion-steel was reached, and consequently the inclusion size distribution is the result of effective collisions and breakage phenomena and the removal of inclusions. Hence, it is expected that estimated PDFs in specimens obtained in later stages of processing can provide information on the size distribution at the end of the liquid steel treatment and its subsequent evolution. This provides evidence of the occurrence of phenomena, such as the reoxidation process, that form new inclusions after liquid treatment. It is worth mentioning recent works that use the PDF approach. Piva and Pistorius [8] used the PDF to show the evolution of the size distribution for different processing routes for steel Ca treatment, while Qifeng et al. [9] used it to analyze results obtained from the modeling of the nucleation, growth, and agglomeration of Al_2_O_3_ inclusions.

On the other hand, the generalized extreme value (GEV) theory based on Murakami et al.’s pioneering work [10] has been used to describe the large inclusion size tail of the size distribution, in which standard probability distributions, such as the log-normal distribution, are insufficient [4]. The method is based on measuring the maximum inclusion size in random inspection areas and fitting the Gumbel distribution to these measurements. This allows to predict the maximum inclusion length (*L_max_*), i.e., the longest inclusion expected to be found in a predetermined area. The fitting of the Gumbel distribution to the size distributions of different specimens enables the statistical comparison and discrimination of *L_max_*. Variations in the procedures used for the application of GEV led to the development of the ASTM E2283 standard “Standard Practice for Extreme Value Analysis of Nonmetallic Inclusions in Steel and other Microstructural Features” [11] as a guide to evaluate inclusion cleanliness. The standard was shown to be a reliable tool to assess inclusion cleanliness. Recently, Kumar and Balachandran [12] showed the standard as an effective tool for estimating the largest inclusion, which has the potential for crack nucleation that leads to fatigue failure in steel specimens. Fuchs et al. [13] confirmed the effectiveness of the standard in the evaluation of inclusion cleanliness in ultraclean gear steels. Furthermore, the standard has been used in the evaluation of the maximum inclusion size of heat-treated wire rod specimens [14]. More recently, the results using the standard showed relatively good agreement with measurements of large inclusions based on the X-ray microcomputed tomography method [15]

In this work, size distributions were measured in steel wire rod specimens from six different plant-scale heats following the metallographic procedure indicated in the ASTM E2283 standard. The PDF approach was used to analyze the whole size distribution, whereas the GEV theory was employed to describe the upper tail of the size distributions following the procedure specified in the ASTM E2283 standard and to estimate the survival probability.

## 2. Background

### 2.1. PDF Approach

The PDF concept, introduced by Higgins [3], is expressed by the following equation:

(1) $$PDF=\frac{n_{v\;}\left(L_{XY}\right)}{\left(L_{Y}-L_{X}\right)}\;\;$$

where $n_{v\;}\left(L_{XY}\right)$ is the frequency of inclusions in a given size bin (particle number per volume), and $\left(L_{Y}-L_{X}\right)\;$ is the bin width with units of length. The logarithmic representation of PDFs can be linear or quadratic and can be described by fractal (power law) and lognormal distributions, respectively [6]. The probability density function of the fractal distribution is given by

(2) $$f\left(x\right)=\frac{C}{x^{D}}$$

where *C* is the constant of proportionality, and *D* is the fractal dimension. The probability density function of the lognormal distribution is

(3) $$f\left(x\right)=\frac{1}{x\;\sigma {\left(2\pi \right)}^{1/2}}\exp\left[\frac{{\left[\ln\left(x\right)-\ln\left(\mu \right)\right]}^{2}}{2\sigma^{2}}\right]$$

where *m* and *s* are the mean and standard deviation, respectively.

### 2.2. ASTM E2283 Standard

The ASTM E2283 standard [11] describes a procedure to statistically characterize the distribution of the largest particles in a solid matrix. It can be used in the case of inclusions in a steel matrix. Herein, essential aspects allowing us to understand the use of the standard in this work are introduced.

The ASTM E2283 procedure is based upon quantitative optical metallographic measurements and their analysis via statistical GEV theory. Six specimens per analysis are required for metallographic measurements. An area of 150 mm^2^ (control area *A_o_*) must be evaluated in four different metallographically prepared planes in each specimen, and the largest measured inclusion in each *A_o_* is recorded. The measurements must be made using the correct magnification to ensure that the detected largest inclusion is a minimum of 20 pixels in length. The procedure provides a dataset of the 24 largest inclusions, which are listed in ascending order and can be represented as *x_i_*. The cumulative probability *P_i_* is calculated by the following equation:

(4) $$P_{i}=\frac{x_{i}}{\left(N+1\right)}\;$$

where *N* = 24.

The prediction of *L_max_* is based on the fitting of the Gumbel extreme value distribution to the 24 recorded inclusion lengths. The probability density function of the Gumbel distribution is expressed by

(5) $$f_{\left(x\right)}=\frac{1}{\delta }\left[exp\left(\frac{x-\lambda }{\delta }\right)\right]\times exp\left[-exp\left(-\frac{x-\lambda }{\delta }\right)\right]$$

where *x* is the random variable (maximum inclusion length), and *l* and *d* are the location and scale parameters, respectively. The corresponding cumulative distribution is

(6) $$F_{\left(x\right)}=exp\left(-exp\left(-\frac{x-\lambda }{\delta }\right)\right)$$

which can be rewritten by introducing the reduced variate *y*:

(7) $$F_{\left(y\right)}=exp\left(-exp\left(-\;y\;\right)\right)$$

where

(8) $$y=\frac{x-\lambda }{\delta }$$

Solving Equation (8) for *x*,

(9) x= δ  y+λ

From Equation (7), *y* can be expressed in terms of the cumulative function as

(10) y=−ln(−ln(F(y)))

Equation (10) can be related to Equation (4), and the expression for *y* is rewritten as

(11) y=−ln(−ln(F(y)))= −ln(−ln(P))

On the other hand, the return period *T* is used to predict how large an inclusion could be expected to be found if a reference area (*A_ref_*) larger than *A_o_* were to be evaluated. That is,

(12) $$T=\frac{A_{ref}}{A_{o}}$$

In the ASTM E2283 standard, *A_ref_* is chosen to be 1000 times larger than *A_o_*_._

Furthermore, *T* is statistically defined as

(13) $$T\;=\;\frac{1}{1-P}$$

For a *T* value of 1000, the corresponding *P* value is 0.999 (99.9%).

Thus, for *P* = 0.999, the $y_{\left(P=0.99\right)}$ value is calculated using Equation (11), and the corresponding *x*_(*P*=0.99)_ value is calculated by Equation (9). The calculated *x*_(*P*=0.99)_ value corresponds to *L_max_*, and Equation (9) is rewritten as

(14) $$x_{\left(P=0.99\right)}=L_{max}=\;\delta \;\;y_{\left(0.99\right)}+\lambda$$

The *δ* and *λ* parameters are calculated using the maximum likelihood method *ML* from the log of the distribution function:

(15) $$LL=\overset{n}{\underset{i=1}{\sum }}ln\left(\frac{1}{\delta }\right)-\left(\frac{x_{i}-\lambda }{\delta }\right)-exp\left(-\frac{x_{i}-\lambda }{\delta }\right)$$

The estimated parameters are referenced as *λ_ML_* and *δ_ML_* and are used to construct the best-fit line through the data points using Equation (9).

The standard error *SE* for any inclusion of length *x* is

(16) $$SE_{x}=\delta_{ML}\sqrt{\left(1.109+0.514y+0.608y^{2}\right)/n}$$

The approximate 95% confidence interval is given by

(17) 95% CI=±2 SE(x)

The comparison of differences in sizes of large inclusions in two steels, denoted *A* and *B*, is calculated by the approximate 95% confidence interval for *L_max_(A)* − *L_max_(B)* by the following equation:

(18) $$C.I=L_{max}\left(A\right)-L_{max}\left(B\right)\pm 2\sqrt{S.E_{ref}{\left(A\right)}^{2}+S.E_{ref}{\left(B\right)}^{2}}$$

If the lower to upper bounds of the 95% *CI* include 0, then it is concluded that there is no difference in the characteristic sizes of the largest inclusions in heats *A* and *B*.

## 3. Materials and Methods

Measurements and analyses of inclusion size distributions in wire rod specimens from six plant-scale heats were conducted. The PDF approach was used to analyze the whole size distribution, and GEV theory was employed to analyze the upper tail of the size distributions.

Specimens from six different heats of high carbon rod wire produced at the plant scale were studied. The heats were produced by an electric arc furnace-ladle treatment-degassing-continuous casting route, and the liquid steel was deoxidized with Si. The studied steel corresponded to SAE 9254, and Table 1 shows the chemical composition expressed in weight percent (wt. %) for two specimens of each heat. The chemical analysis of C and S was performed using the infrared combustion technique, while the content of other elements was determined using the spark emission spectroscopy technique.

**Figure 1:**
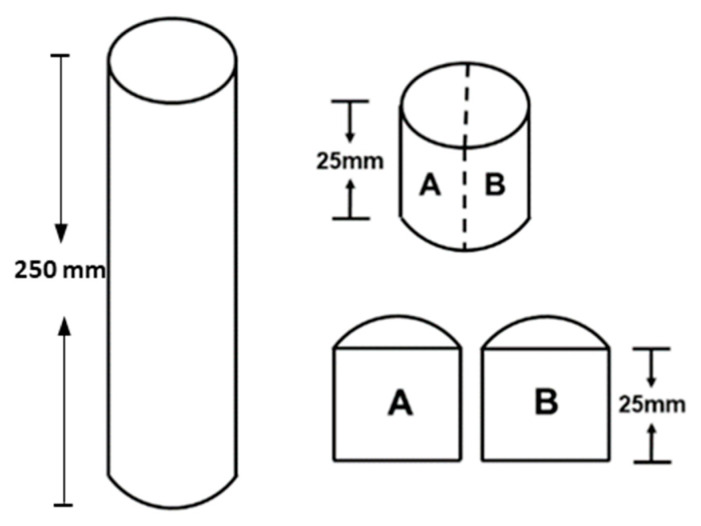
Obtention of probes for metallographic measurements.

Inclusion size distributions were estimated using the metallographic procedure detailed in the ASTM E2283 standard, which was rigorously followed. For each heat, six specimens of wire rod in as-rolled conditions of 25 mm diameter and 200 mm length were available. Each specimen was cut into a probe of 25 mm length and sectioned longitudinally as shown in Figure 1. The plane denoted as A was progressively dry ground using 80-, 220-, 300-, 500-, 800-, 1000-, and 1200-grit SiC paper and polished with 3 and 1 μm diamond paste. The measurements were conducted under a Nikon Eclipse MA200 light optical microscope (Minato ku, Japan) at 100X magnification on 150 fields by image analysis, and the total analyzed surface was 150 mm^2^. Three additional planes were analyzed, ensuring that the space between neighboring planes was at least 0.3 mm, thus avoiding inclusions being counted more than once. The total number of analyzed fields per heat was 600.

The totality of the analyzed inclusions in each heat was considered to estimate the corresponding PDF using Equation (1). The frequency of inclusions per volume unit specified in that equation was estimated from the two-dimensional inclusion measurements obtained by image analysis, which were transformed into three-dimensional data using a procedure based on the Saltikov method [16,17].

By applying the ASTM E2283 standard, the measured maximum inclusion size in each of the inspected planes (24) was used for the statistical analyses described in the standard and synthetized in the previous section of this paper.

## 4. Results and Discussion

**Figure 2:**
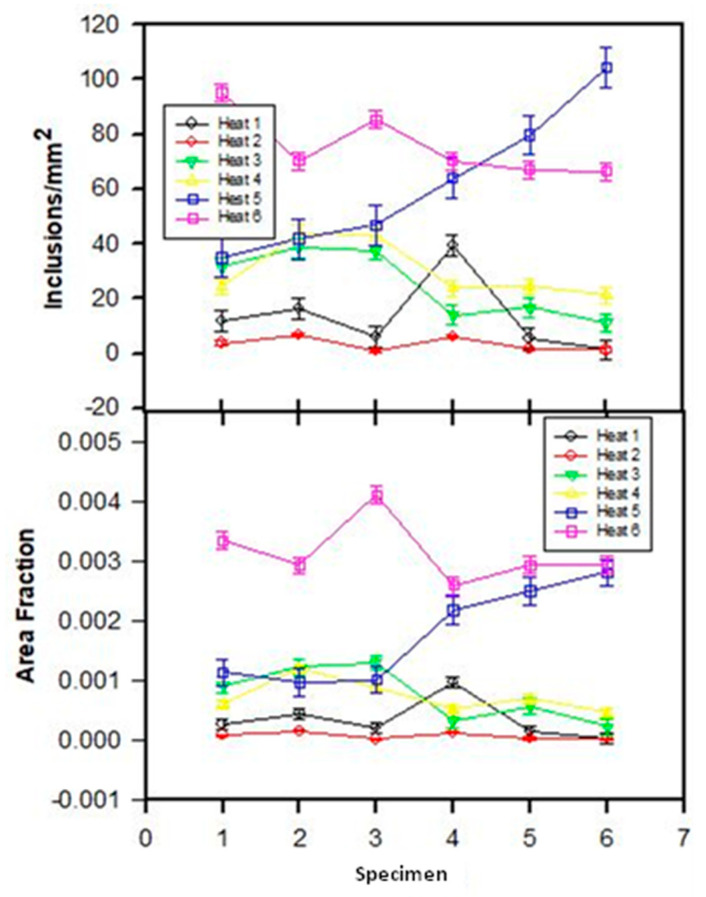
Evolution of inclusions/mm^2^ and area fraction of inclusions.

Figure 2 shows the measured inclusion frequency (inclusions/mm^2^) and area fraction for the six specimens of each heat. In general, Heats 5 and 6 presented the highest values for both parameters, whereas Heats 1 and 2 presented the lowest values. Furthermore, Heats 5 and 6 showed more variability among specimens of the values of both parameters.

### 4.1. Population Density Function PDF

**Figure 3:**
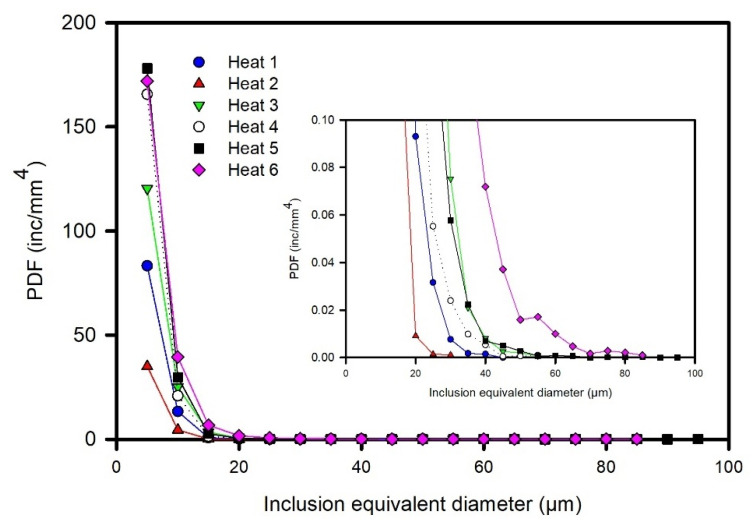
Evolution of the population density function PDF.

Figure 3 shows the calculated PDF for each heat. The inclusion size is referred to as the inclusion equivalent diameter, which is defined as the diameter of a circle with the same area as the recorded particle [5]. Great differences were observed at small inclusion sizes, which decreased as the inclusion size increased. Below 10 μm, Heat 2 presented the lowest PDF values and was followed by Heats 1 and 3, while the rest of the heats presented similar values. In the range of 10–20 μm, Heat 2 continued to present the lowest values, followed by Heat 1, whereas Heat 6 presented the highest values; the rest of the heats presented similar values. Furthermore, the magnification of the PDF scale (inner figure) shows that at larger inclusion sizes, differences are also observed in PDF values and that Heats 2 and 6 showed the lowest and highest values of PDF, respectively. Thus, it can be stated for the studied heats that as the frequency of small inclusions increased, the population of large inclusions also increased, i.e., that the presence of large inclusions was due to the processing of liquid steel rather than to an eventual phenomenon.

**Figure 4:**
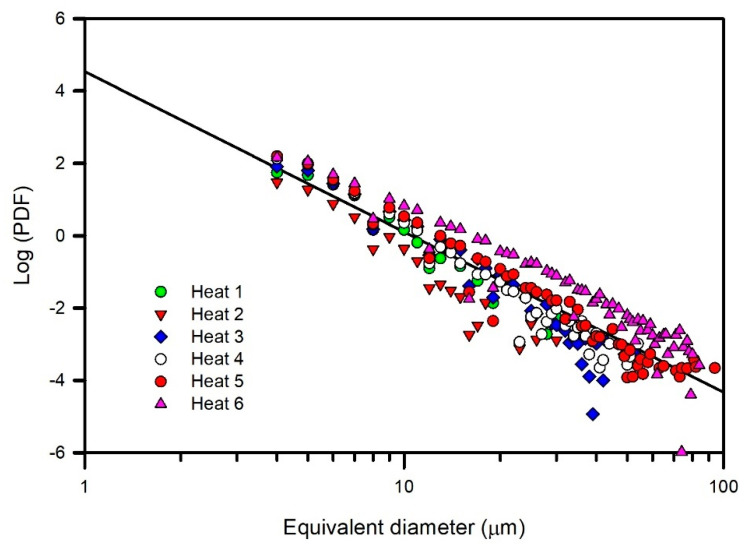
Log-log plots of PDFs versus inclusion equivalent diameter for all heats.

**Figure 5:**
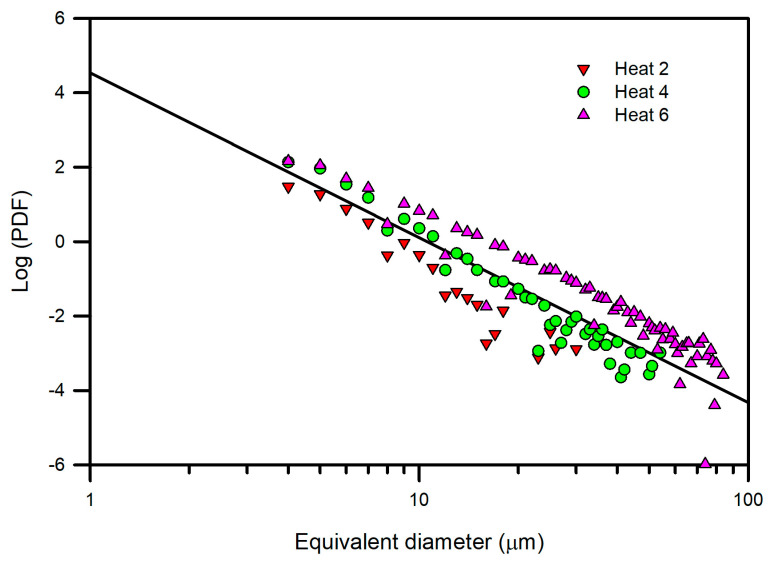
Log-log plot of PDFs versus inclusion equivalent diameter for Heats 2, 4 and 6.

**Figure 6:**
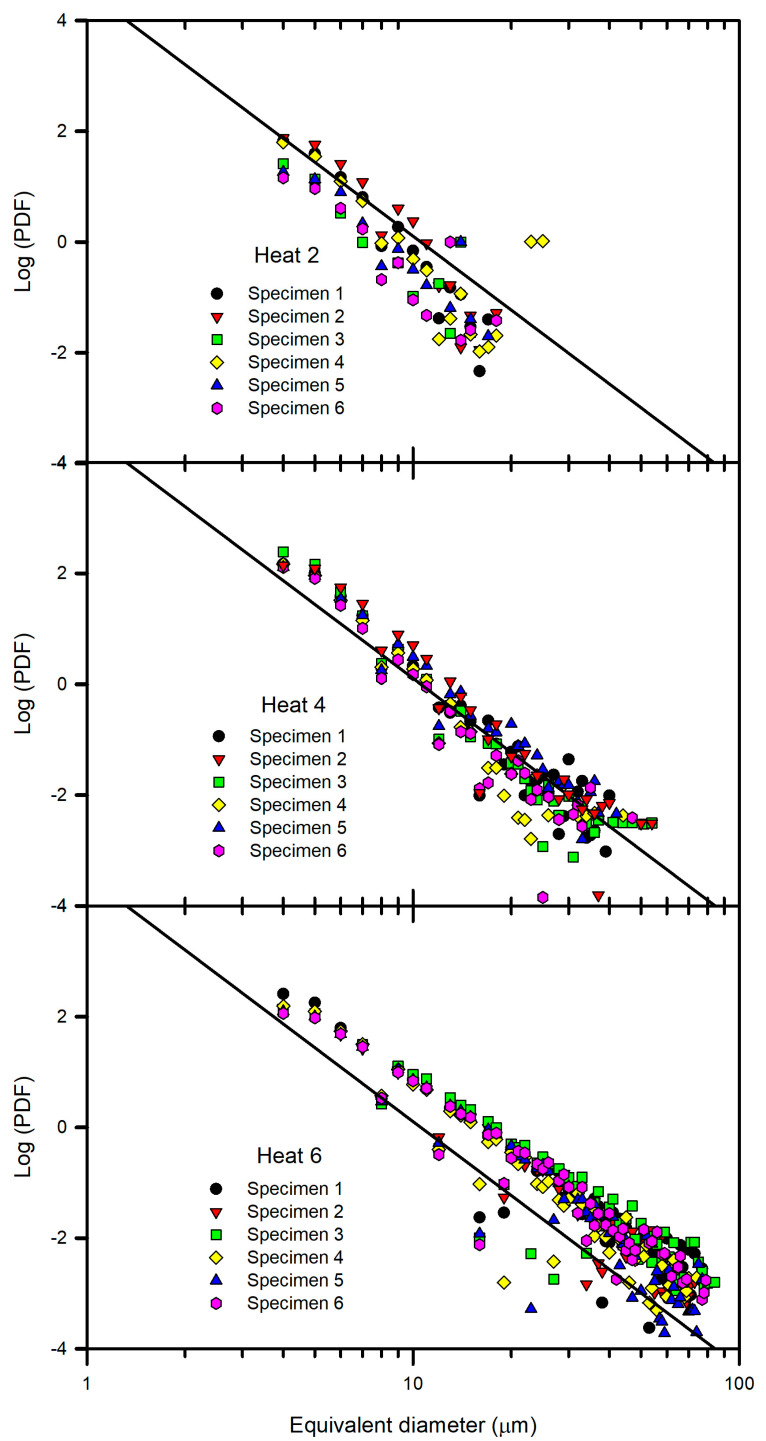
Log-log plot of PDFs versus inclusion equivalent diameter for specimens of Heats 2, 4 and 6.

The logarithmic representation of the calculated PDFs is shown in Figure 4. A general linear power law behavior, represented by a reference straight line plotted in the figure, was observed. The reference straight line was estimated from the totality of the PDF data. The most notable deviations from the reference line corresponded to Heats 2 and 6, respectively, whereas the other heats fit better to the reference line. To clarify the observed behavior, the logarithmic representations of the PDFs of Heats 2 and 6 are shown in Figure 5, in which the data of Heat 4 were included because of its better fit to the reference line. Heat 6 presented both a higher frequency of inclusions and a larger inclusion size; in contrast, Heat 2 presented a lower frequency of inclusions and a smaller inclusion size, and Heat 4 exhibited intermediate values of both parameters. To individually analyze the heats of Figure 5, the data corresponding to the associated six samples of each heat are shown in Figure 6. As expected from a statistical point of view, more dispersion between the data were observed as the frequency decreased (Heat 2), although the linear trend continued to be observed. Thus, the heats can be ordered in decreasing order of cleanliness as follows: Heat 2, 4 and 6. Furthermore, Heats 3 and 5 had a similar behavior to Heat 4.

**Figure 7:**
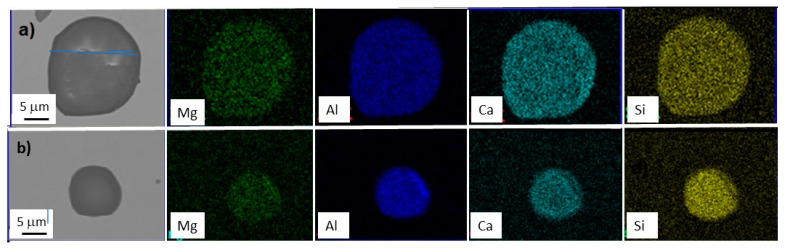
Elemental mapping for SiO_2_-Al_2_O_3_-CaO-MgO inclusions of different sizes: (**a**) 20 μm and (**b**) 12 μm.

**Figure 8:**
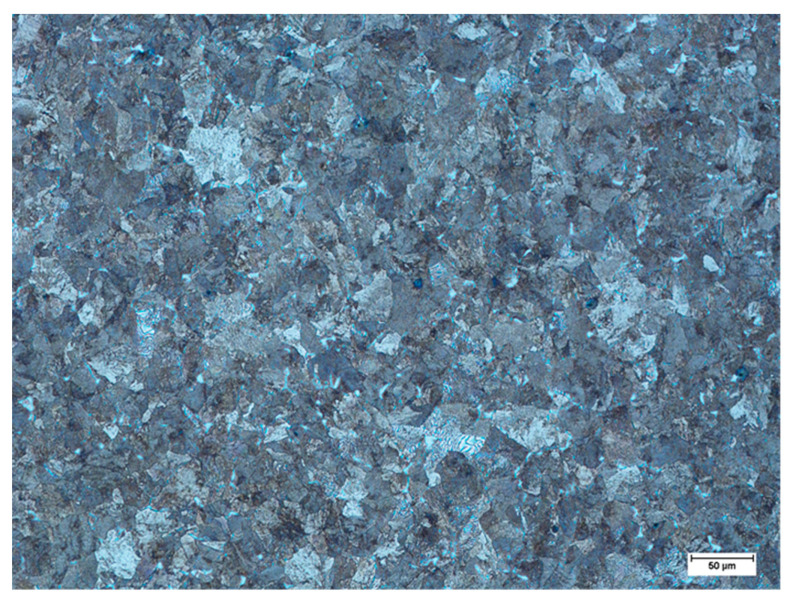
Typical microstructure of the steel matrix for the studied heats.

Then, the differences between the slopes of the straight lines of each heat can be deduced. The slope of the straight line is associated with the refining process and is specific to each process. Van Ende et al. [5] showed results for Ti-alloyed Al-killed steel produced in different plants. At the end of the refining process, a linear behavior of log PDF with a good fit was shown, and the slope values of the straight line were similar regardless of the plant of production. The slope value (−3.5) was associated with the Al-deoxidation process. Thus, the average slope value of the reference line (−0.089) in Figure 4 can be associated with the Mn–Si deoxidation process. In previous work [18], SiO_2_-Al_2_O_3_-MnO inclusions were observed at the beginning of the ladle treatment, which evolved to SiO_2_-Al_2_O_3_-CaO-MgO inclusions during treatment. Of note, SiO_2_-Al_2_O_3_-CaO-MgO inclusions were observed in this work under as-rolling conditions, as shown in Figure 7 for inclusions of different sizes. This result suggests that the estimated slope of the reference line is associated with the formation of SiO_2_-Al_2_O_3_-MnO inclusions during the Mn-Si deoxidation process and their evolution to SiO_2_-Al_2_O_3_-CaO-MgO inclusions during ladle treatment. Furthermore, the linear behavior indicates that the new inclusions were not formed after liquid steel treatment and that inclusion populations evolved by the growth of inclusions, breakage, and removal of inclusions. In addition, the inclusions found in all samples were embedded in a matrix similar to that presented in Figure 8. The microstructure of the matrix did not vary due to the similarity of the chemical composition (Table 1) and because the inclusions were subjected to the same thermomechanical treatment.

### 4.2. Extreme Value Distribution Analysis

**Figure 9:**
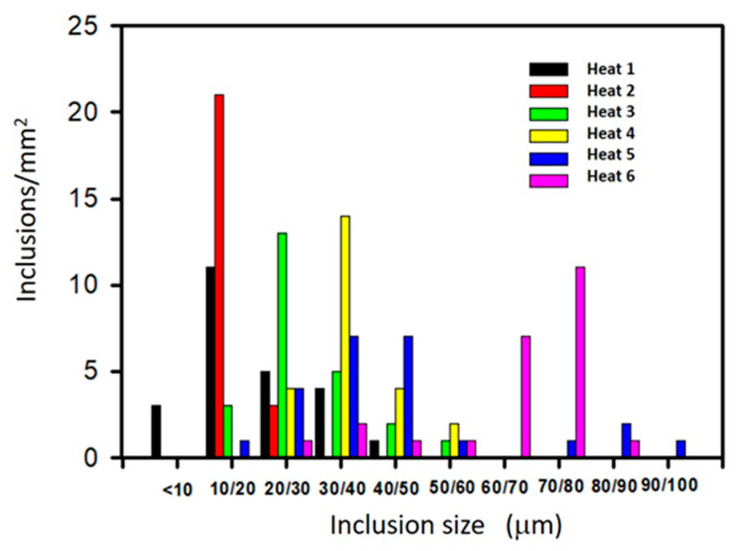
Inclusion size distribution.

Figure 9 shows a representation of the inclusion size distribution in histogram format. In this section, “length inclusion” is used to denote the inclusion size, just as the ASTM E2283 standard does. Of note, the inclusion length corresponds to the “equivalent diameter” used in the previous section. Two main observations can be highlighted. Below 20 μm, the highest frequency was shown by Heat 2; above 70 μm, however, Heat 6 had the highest frequency. For intermediate inclusion sizes, it is difficult to describe a trend.

**Figure 10:**
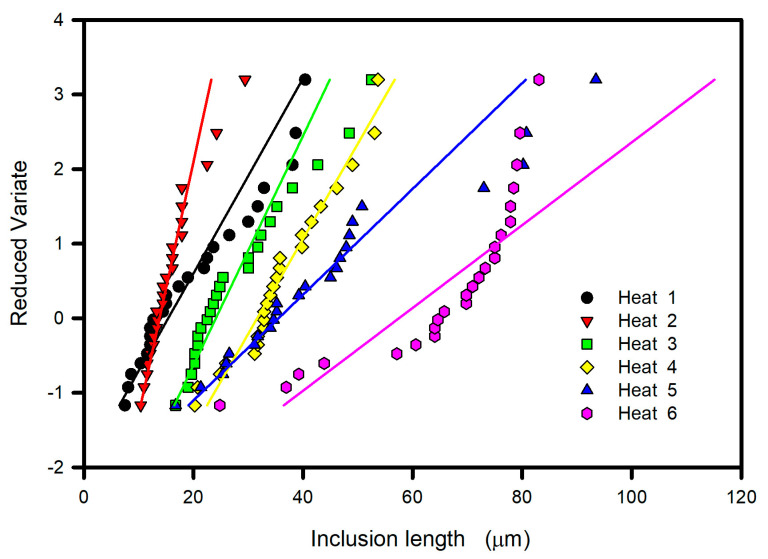
Reduced variate versus inclusion length.

Table 2 shows the 24 measured values of maximum inclusion length obtained for each heat from four analyzed planes in each of the six available specimens. The maximum inclusion length values are listed in increasing order as specified by the ASTM E2283 standard. The reduced variate was calculated using Equation (11) and is shown in Figure 10 for each heat as a function of the inclusion length. In general, a linear behavior for individual heats was observed. The fit of the straight lines was better at small inclusion lengths, and scattering was observed at longer inclusion lengths. It is also observed that steeper slopes correspond to lines located at shorter inclusion lengths, and consequently, the interception with the horizontal axis was different for each line.

**Figure 11:**
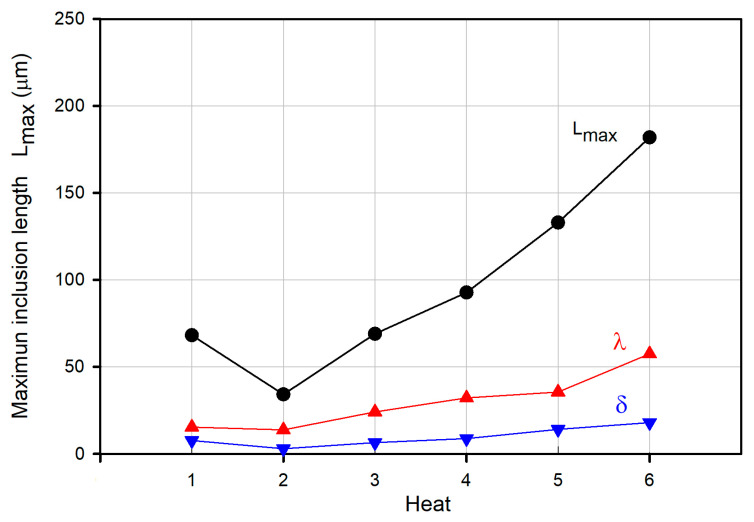
Maximum inclusion size *L_max_*, *λ*, and *δ* for all heats.

**Figure 12:**
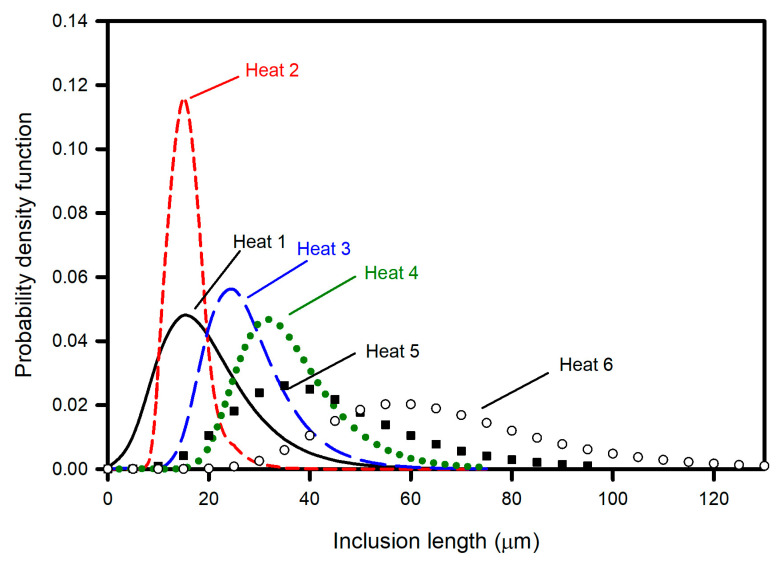
Probability density function for heats.

Table 3 lists the values of the estimated statistical parameters according to the procedure of the ASTM E2283 standard. The values of *λ*, *δ*, and *L_max_* are plotted in Figure 11, in which it is observed that the lower the values of *λ* and *δ* are, the lower are the values of *L_max_*. Thus, the heats can be listed in increasing order of *L_max_* values as follows: Heat 2, Heat 1, Heat 3, Heat 4, Heat 5, and Heat 6. This behavior is illustrated in Figure 12, where the probability distribution functions calculated via Equation (5) and using the values of *λ* and *δ* included in Table 3 are plotted.

To determine whether the observed differences in *L_max_* values were statistically significant, *L_max_* values were compared according to the procedure described in the ASTM E2283 standard. The criterion of a 95% C.I. was calculated from the predicted value of *L_max_* for each heat as well as the corresponding standard error. Table 4 lists the results of a round-robin comparison in matrix format, where the column and arrow titles correspond to the identification of heats. The interception of a column with an arrow denotes the range of bounds of the 95% C.I. of the corresponding heats. A C.I. range that does not include 0 is denoted in red and indicates a difference between the compared *L_max_*; otherwise, it is denoted in green and indicates that there was no difference. The *L_max_* of Heats 5 and 6 did not differ from each other; in contrast, they differed from the *L_max_* of the rest of the heats. Furthermore, *L_max_* of Heat 2 was different from other *L_max_*, and *L_max_* of Heats 1, 3 and 4 were not different from each other. Thus, using the *L_max_* parameter as the cleanliness index, it can be stated that Heat 2 is the cleanest and significantly different from the rest of the heats; Heats 5 and 6 are the worst and significantly different from the rest of the heats; Heats 1, 3 and 4 are not different from each other, and their inclusion cleanliness is intermediate between Heat 2 and Heats 5 and 6.

**Figure 13:**
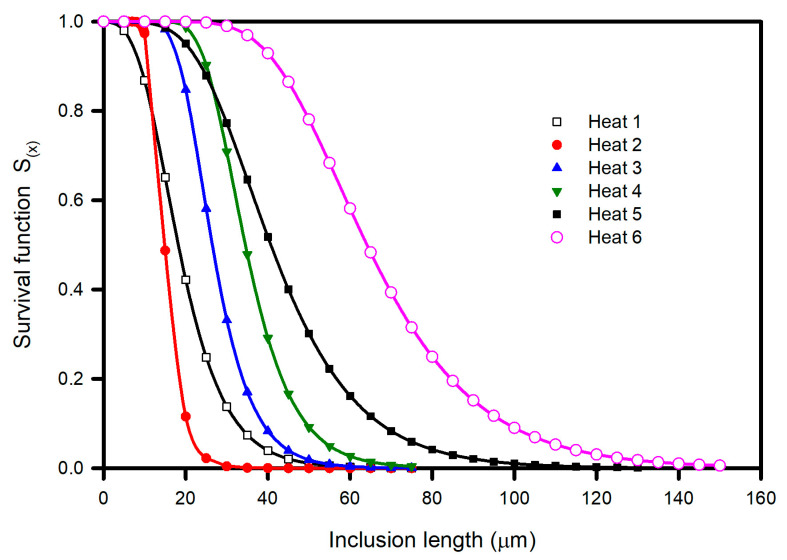
Survival function S_(x)_ for each heat.

Although *L_max_* represents a useful indicator that enables the comparison and discrimination of inclusion populations, there is often interest in knowing the probability of finding inclusions greater than a certain “critical” size depending on the specific application of the steel component. For example, in components subjected to fatigue, it is accepted that the size of the inclusion is important because cracks can form at the inclusion–steel interface. In this context, the survival function S_(x)_ (probability of finding an inclusion larger than a given length, equal to the complement of the cumulative density function) rather than *L_max_* is more convenient. Figure 13 presents S_(x)_ as a function of the inclusion length for all heats. The order of heats previously stated as a function of *L_max_* is confirmed for inclusion lengths greater than 35 μm, where no overlapping of curves is observed. At 35- and 10 μm inclusion lengths, the curves of Heats 3 and 4 and Heats 1 and 2 overlapped, and therefore, the order of heats was modified.

**Figure 14:**
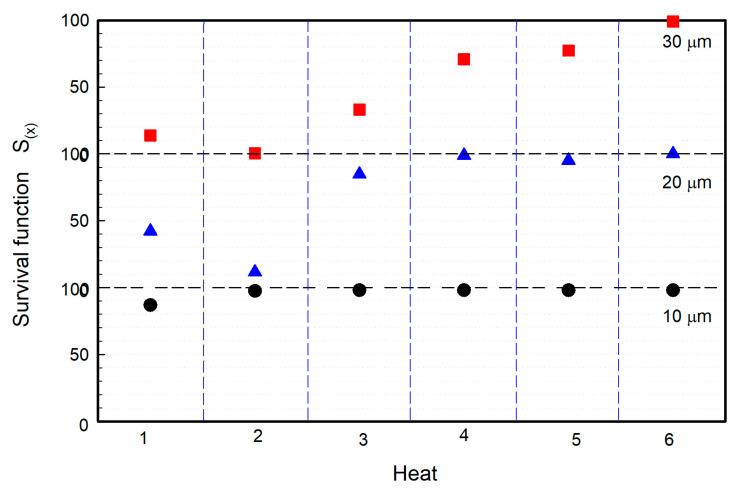
Survival function S_(x)_ for different “critical” inclusion sizes.

S_(x)_ is plotted in Figure 14 from the data of Figure 13 for three critical inclusion lengths: 10, 20 and 30 μm. The heats can be ordered by increasing the S_(x)_ value for each critical value; for example, for an inclusion length shorter than 10 μm, the order is Heat 1, Heat 2, Heat 3, Heat 4, Heat 5 and Heat 6. This order is altered when the critical value of 20 μm is selected, changing the order to Heat 2, Heat 1, Heat 3, Heat 5, Heat 4 and Heat 6. The observed difference is explained by the overlapping probability density curves and S_(x)_ curves for Heats 1 and 2 and for Heats 4 and 5. For example, in Heat 1 and Heat 2, overlapping can be observed in the upper left corner in Figure 13, or the overlapping of probability density functions in the lower left corner in Figure 11. The same explanation can be used for the inversion of order between Heats 4 and 5. Furthermore, for critical values greater than 35 μm, where no overlapping of curves is observed, the order is Heat 2, Heat 1, Heat 3, Heat 4, Heat 5 and Heat 6. This is the same when parameter *L_max_* is considered as the cleanliness parameter.

## 5. Conclusions

Inclusion size distributions in wire rod specimens from six plant-scale heats were measured and analyzed. The measurements were taken following the metallographic procedure specified in the ASTM E2283 standard. The analysis of size distributions was developed using two approaches: (1) the estimation of PDFs according to the Higgins formalism [3] to obtain information on the whole inclusion size distribution and (2) the use of the extreme value theory to describe the upper tail of the size distributions. Thus, the following conclusions can be drawn.

- Heats were listed in decreasing order of inclusion cleanliness based on the analysis of the linear logarithmic representation of PDFs.
- No new inclusions were formed after the ladle treatment process, as inferred from the linear behavior of the logarithmic representation of PDFs, a power-law-type with time. Hence, the evolution of inclusion distribution was associated with growth, breakage, and the removal of inclusions.
- Heats were listed in decreasing order of inclusion cleanliness using the maximum inclusion length parameter *L_max_*. The use of the extreme value statistics procedure specified in the ASTM E2283 standard led to a statistical comparison of *L_max_*.
- Heats were ordered by considering the survival function S_(x)_ values (probability of finding an inclusion larger than a “critical” inclusion length) estimated using the GEV theory. It was shown that the order can change depending on the critical value.

## Figures and Tables

**Table 1:** Chemical composition (wt. %) of the studied heats.

Heat	Specimen	Content (wt. %)					
		C	Mn	Si	Cr	P	S
1	1	0.57	0.70	1.45	0.66	0.008	0.014
	2	0.58	0.70	1.44	0.67	0.007	0.012
2	1	0.54	0.68	1.43	0.65	0.008	0.014
	2	0.57	0.70	1.45	0.66	0.007	0.014
3	1	0.64	0.71	1.51	0.71	0.009	0.019
	2	0.65	0.72	1.52	0.71	0.009	0.019
4	1	0.59	0.70	1.5	0.70	0.008	0.019
	2	0.60	0.71	1.48	0.69	0.008	0.019
5	1	0.60	0.70	1.5	0.66	0.007	0.17
	2	0.58	0.71	1.52	0.70	0.008	0.17
6	1	0.54	0.71	1.52	0.71	0.009	0.15
	2	0.61	0.70	1.49	0.69	0.008	0.15

**Table 2:** Inclusion maximum length *L_max_* (μm) for analyzed plans in each heat.

N° Inclusion	Heat					
	1	2	3	4	5	6
**1**	7.5	10.4	16.7	20.2	16.7	24.8
**2**	8.1	11.0	19.0	20.8	21.4	36.9
**3**	8.7	11.5	19.6	24.8	25.4	39.2
**4**	10.4	11.5	20.2	26.0	26.0	43.9
**5**	11.6	12.1	20.2	31.2	26.5	57.1
**6**	12.1	12.7	20.8	31.7	31.2	60.6
**7**	12.1	12.7	20.8	31.7	31.7	64.0
**8**	12.1	13.3	21.4	32.9	34.0	64.0
**9**	12.7	13.3	22.5	32.9	34.6	64.6
**10**	14.4	13.3	23.1	32.9	35.2	65.8
**11**	15.0	14.4	23.7	33.45	35.2	69.8
**12**	15.0	14.4	24.2	34.0	39.2	69.8
**13**	17.3	14.4	24.8	34.6	40.4	71.0
**14**	19.0	15.0	25.4	35.2	45.0	72.1
**15**	21.9	16.2	30.0	35.8	46.2	73.3
**16**	22.5	16.2	30.0	35.8	46.7	75.0
**17**	23.7	16.2	31.7	39.8	47.9	75.0
**18**	26.5	17.9	32.3	39.8	48.5	76.2
**19**	30.0	17.9	34.0	41.5	49.0	77.9
**20**	31.7	17.9	35.2	43.3	50.8	77.9
**21**	32.9	17.9	38.1	46.2	73.0	78.5
**22**	38.1	22.6	42.7	49.0	80.2	79.0
**23**	38.7	24.2	48.5	53.1	80.8	79.6
**24**	40.4	29.4	52.5	53.7	93.5	83.1

**Table 3:** Values of statistical parameters (μm).

Parameters	Heat					
	1	2	3	4	5	6
*λ*	15.4	13.8	24.1	32.2	35.6	57.5
*δ*	7.6	2.9	6.5	8.8	14.1	18.0
*L* average	20.0	15.7	28.2	35.8	44.1	65.8
Standard deviation (Sdev)	10.4	4.6	9.6	11.8	19.8	15.3
Maximum length (*L_max_*)	68.2	34.2	69.0	92.7	132.9	181.9
Minimum length Y(*L_min_*)	6.9	6.9	6.9	6.9	6.9	6.9
Standard error (S.E.)	8.9	3.5	7.7	10.4	16.7	21.3

**Table 4:** Range of bounds of 95% confidence interval C.I.

	Heat 2	Heat 3	Heat 4	Heat 5	Heat 6
**Heat 1**	53.23	22.82	2.87	−26.84	−67.43
	14.79	−24.40	−51.96	−102.63	−159.96
**Heat 2**		−17.89	−36.64	−64.63	−104.49
		−51.70	−80.46	−132.87	−190.94
**Heat 3**			2.10	−27.18	−67.56
			−49.60	−100.72	−158.26
**Heat 4**				−0.87	−41.71
				−79.52	−136.60
**Heat 5**					5.21
					−103.14
